# TRPM8 Mutations Associated With Persistent Pain After Surgical Injury of Corneal Trigeminal Axons

**DOI:** 10.1212/NXG.0000000000200206

**Published:** 2024-11-14

**Authors:** Mohammad-Reza Ghovanloo, Philip R. Effraim, Sidharth Tyagi, Alecia M. Aldrich, Xiaoyang Cheng, Jun-Hui Yuan, Betsy R. Schulman, Deborah S. Jacobs, Sulayman D. Dib-Hajj, Stephen G. Waxman

**Affiliations:** From the Department of Neurology (M.-R.G., S.T., A.M.A., X.C., J.-H.Y., B.R.S., S.D.D.-H., S.G.W.), Yale University School of Medicine, New Haven; Center for Neuroscience and Regeneration Research (M.-R.G., P.R.E., S.T., A.M.A., X.C., J.-H.Y., B.R.S., S.D.D.-H., S.G.W.), Yale University, New Haven; Neuro-Rehabilitation Research Center (M.-R.G., P.R.E., S.T., A.M.A., X.C., J.-H.Y., B.R.S., S.D.D.-H., S.G.W.), Veterans Affairs Connecticut Healthcare System, West Haven; Department of Anesthesiology (P.R.E.), Yale University School of Medicine, New Haven, CT; and Department of Ophthalmology (D.S.J.), Massachusetts Eye and Ear, Harvard Medical School, Boston.

## Abstract

**Background and Objectives:**

Despite extensive efforts, the mechanisms underlying pain after axonal injury remain incompletely understood. Pain following corneal refractive surgery offers a valuable human model for investigating trigeminal axonal injury because laser-assisted in situ keratomileusis (LASIK) severs axons of trigeminal ganglion neurons innervating the cornea. While the majority of patients are pain-free shortly after surgery, a minority endure persistent postoperative ocular pain. Through genomic analysis of patients experiencing persistent postoperative ocular pain, we identified rare variants in genes encoding ion channels and receptors, including TRPM8, which codes for the menthol-sensitive and cold-sensing transient receptor potential cation channel.

**Methods:**

We conducted a profiling of 2 TRPM8 mutant variants, D665N and V915M, which were identified in patients suffering from persistent pain after LASIK surgery. We used patch-clamp and multielectrode array (MEA) recordings to investigate the biophysical and pharmacologic properties of mutant vs wild-type (WT) channels.

**Results:**

Patch-clamp analysis shows that these mutations shift the activation curves of TRPM8 in a hyperpolarized direction, with this effect being significantly different between WT and D665N channels. In addition, both mutations significantly increase channel sensitivity to the canonical ligand, menthol. MEA recordings from transfected rat trigeminal ganglion neurons indicate that expression of D665N and V915M mutant channels increases spontaneous activity compared with WT channels. Consistent with patch-clamp results, neuronal activity in MEA recordings was increased on exposure to menthol.

**Discussion:**

Collectively, our findings suggest that proexcitatory mutations of TRPM8, in the context of axonal injury within the cornea, can produce trigeminal ganglion neuron hyperexcitability that contributes to persistent postoperative ocular pain. In addition to providing additional evidence for a role of TRPM8 in human pain, our results suggest that inhibitors of this channel merit future study.

## Introduction

The mechanisms underlying pain following axonal injury are incompletely understood. In particular, there is a need to understand why one individual will develop severe pain after nerve injury, while other individuals do not experience pain following similar injuries. Pain arising from corneal refractive surgery provides an opportunity to investigate pathophysiologic mechanisms through which axonal injury affects trigeminal neurons.^1-4^ One common type of corneal refractive surgery, laser-assisted in situ keratomileusis (LASIK), involves relatively reproducible axonal transection within the cornea. Although most patients experience only a short-term pain after LASIK, some patients experience severe chronic pain.^1-3^

The pathophysiologic mechanisms that underlie this persistent postoperative pain are not well understood. To gain a better understanding of mechanistic basis of persistent pain after LASIK, we have examined genetic factors that might predispose carriers for this postoperative pain. We previously performed whole-exome sequencing (WES) in 21 patients, who reported severe chronic pain after LASIK, and identified multiple mutant variants in genes that comprise the electrogenisome (the set of genes that confer electrical excitability) and the associated electroproteome (set of proteins that confer electrical excitability) of trigeminal ganglion neurons.^5,6^ These genes encode several voltage-gated sodium (Nav) channels, calcium channels, potassium (Kv) channels, and transient receptor potential (TRP) channels.^5^

An increase in the activity of channels that comprise the electrogenisome would be expected to enhance excitability of trigeminal neurons, which could contribute to postsurgical pain. We recently characterized the biophysical properties of a mutant variant of Nav1.7, P610T, that was discovered in 2 siblings with post-LASIK pain, and showed that it causes an impairment in slow inactivation; this gain-of-function mutation in Nav1.7 produces hyperexcitability of trigeminal neurons, which may contribute to persistent ocular pain after LASIK.^4^

Here, we focus on 2 mutations in TRPM8, D665N, and V915M, that were discovered in our cohort of patients with persistent pain following corneal refractive surgery.^5^ TRPM8 channels can be activated by natural and synthetic cooling agents, including menthol, and their role in sensing cooler temperatures is well-established.^7^ TRPM8 has diverse physiologic roles. This channel is expressed in about 15% of all somatosensory neurons, which include small diameter and unmyelinated C-fibers and lightly myelinated Aδ fibers.^7-10^ Although multiple studies have implicated TRP channels in pain, only a few studies have examined the role of TRPM8 in human pain disorders. Two studies have shown that activation of TRPM8 can exacerbate pain sensation,^11,12^ and we recently characterized a gain-of-function mutant in TRPM8 in a patient with trigeminal neuralgia.^13^ TRPM8 has also been indicated as a key contributor to the physiology of the eye, including tear production and cold thermoreception that triggers basal tear flow.^14-19^

In this study, our goal was to determine whether 2 mutant variants in TRPM8, D665N, and V915M, that were discovered in patients with persistent ocular pain after corneal refractive surgery, produce any changes in the biophysical and pharmacologic properties of TRPM8, and on the excitability of trigeminal ganglion neurons. Our patch-clamp and MEA recordings demonstrate that these mutations indeed produce gain-of-function effects, suggesting a mechanism by which they contribute to the painful phenotypes in our patients.

## Methods

### Standard Protocol Approvals, Registrations, and Patient Consents

Written consents were obtained from all participants and approved by Yale Human Investigation Committee (0608001728). Animal studies followed a protocol approved by the Department of Veterans Affairs West Haven Hospital Institutional Animal Care and Use Committee.

### Mutation Ascertainment

These 2 patients were identified from a series of 21 cases, who developed persistent (>3 months) and moderate-to-severe corneal pain (Ocular Surface Disease Index, OSDI score >12) after LASIK surgery, in a study approved by the Yale Human Investigation Committee.^5^ A 4-step quality-based variant filtering was used: (1) sequencing read depth ≥10-fold, (2) variant allele frequency >20%, (3) genotype quality ≥20, and (4) passed GATK quality filters. Variants with allele frequency ≥0.05 in any of the annotated databases were removed. Whole-exome sequencing of genomic DNA was performed with 98.4% of the target regions with mean coverage of 10X or higher, and low-frequency variants were identified.^5^

### Cell Culture

Human embryonic kidney (HEK-293) cells (ATCC, Manassas, VA, USA CLS Cat# 300192/p777_HEK293, RRID:CVCL_0045) were used for automated patch-clamp experiments. The plasmid carrying the human TRPM8 cDNA fused in-frame with 2A-GFP was previously described.^13^ The missense mutations were introduced using QuikChangeII XL site-directed mutagenesis (Stratagene). HEK-293 cells were transiently transfected with human TRPM8 plasmids using Lipofectamine 2000 using a total of 10 µg cDNA/transfection. All experiments were head-to-head comparisons with wild-type (WT) referring to cells transfected with WT channels, and D665N and V915M refer to respective cells transfected with mutant channels. All cells were incubated at 37°C/5% CO_2_, as described in reference 4.

Experiments were performed on sorted cells. Cells at a density of ∼3 × 10^6/mL were transfected with TRPM8 channel constructs (tagged with 2A-GFP). Transfection-enhancing solution was added to cell suspensions 24 hours after plasmid addition as per the manufacturer protocol. Cells were harvested for fluorescence-activated cell sorting (FACS) after incubation for an additional 24 hours. FACS was conducted using the Wolf G2 NanoCellect cell sorter (Nanocollect Biomedical Inc., San Diego, CA) with GFP-positive–sorted cells resuspended in ExCell media. A minimum of 500,000 cells were collected in each condition. After FACS, samples were manually inspected to ensure that >95% of cells were positive for green fluorescence.

### Automated Patch-Clamp

We used automated patch-clamp recording for all HEK-293 experiments. The protocols we used are based on the following studies.^20,21^ TRPM8 currents were measured in the whole-cell configuration using a Qube-384 (Sophion A/S, Copenhagen, Denmark)–automated patch-clamp system. Intracellular solution contained (in mM) 140 CsF, 0.3 CaCl_2_, 2 MgATP, 10 EGTA, and 10 HEPES, adjusted to pH7.2 with CsOH. The extracellular recording solution contained (in mM) 150 NaCl, 5 KCl, 1 MgCl_2_, 1.8 CaCl_2_, 10 glucose, and 10 HEPES, adjusted to pH7.4 with NaOH.^13^ Liquid junction potentials calculated to be ∼7 mV were not adjusted for. Currents were low pass filtered at 5 kHz and recorded at 25 kHz sampling frequency. Series resistance compensation was applied at 100%, and leak subtraction enabled. The Qube-384 temperature controller was used to maintain recording chamber temperature for all experiments at 22 ± 2°C. Appropriate filters for resistances were used. Data analysis was performed, based via the same methodology that we developed in reference 4, using Analyzer (Sophion A/S, Copenhagen, Denmark) and Prism (GraphPad Software Inc., La Jolla, CA) software. All patch-clamp experiments were performed using the Qube. The biophysical characterization of WT vs mutant channels was performed simultaneously on the same Q-Chips. Menthol was purchased from Sigma and prepared in DMSO for stock.

### Activation Protocols

To determine the voltage dependence of activation, we measured the peak current amplitude at test pulse potentials ranging from −100 mV to +240 mV in increments of +5 mV for 100 ms from a holding potential of −60 mV. The protocol we used was based on the work of reference 22. Channel conductance (G) was calculated from peak I:

(1) G=I/(V−E)

where G is the conductance, I is the peak current in response to the command potential V, and E (measured on IV relationships) is the Nernst equilibrium potential. Calculated values for conductance were fit with the Boltzmann equation:

(2) $${G/G}_{\max}=1/\left(1+\exp\left[V_{1/2}-V_{m}\right]/k\right)$$

where G/G_max_ is the normalized conductance amplitude, V_m_ is the command potential, V_1/2_ is the midpoint voltage, and k is the slope. The calculations and curve fitting were based on our previously published work in Refs. 4,21.

### Multielectrode Array

The methodologies and analyses that we used here were based on our previously published work in Refs. 4,21,23. Details: each multielectrode array (MEA) experiment was performed in at least 3 biological replicates. For each replicate, trigeminal ganglion neurons were isolated from 3 P4 Sprague-Dawley rat pups (6 trigeminal ganglia in total) in the same manner as previously described.^24^ After isolating the trigeminal ganglion neurons, they were incubated in oxygenated complete saline solution (CSS) (which comprises 137 mM NaCl, 5.3 mM KCl, 1 mM MgCl_2_, 25 mM sorbitol, 3 mM CaCl_2_, and 10 mM HEPES, pH 7.2 adjusted with NaOH) and supplemented with 0.5 U/mL Liberase TM (Roche, Basel, Switzerland) and 0.6 mM EDTA for 20 minutes at 37°C. The tissue was subsequently incubated for 15 minutes at 37°C in CSS with 0.5 U/mL Liberase TL (Roche), 0.6 mM EDTA, and 30 U/mL papain (Worthington Biochemical). Neurons were pelleted (100×*g* for 3 minutes) and triturated in 0.5 mL of culture medium containing 1.5 mg/mL low endotoxin BSA and 1.5 mg/mL trypsin inhibitor (Sigma, St. Louis, MO).

Following the second digestion and pelleting, transfection was accomplished via electroporation with 2 µg of plasmid encoding either WT or the TRPM8 variants using Nucleofector IIS (Lonza, Basel, Switzerland) and Amaxa Basic Neuron SCN Nucleofector Kit (VSPI-1003) using protocol SCN-BNP 6. Typically, 25–30% of neurons are transfected. Recovering the neurons was performed in 100 μL of Ca-free DMEM for 5 minutes at 37°C, followed by dilution of the cell solution with culture medium supplemented with 50 ng/mL nerve growth factor and 50 ng/mL glial cell line–derived neurotrophic factor. Neurons were then seeded onto MEA plates which had been precoated with poly-d-lysine and laminin. Each experimental condition was seeded into 6 wells of a 24-well MEA plate, with each well containing 16 electrodes in a 4 × 4 array. The trigeminal neurons were at 37°C for 45 minutes in a 5% CO_2_ incubator to allow the neurons to attach. Following this, they were fed with 500 μL of culture media and incubated at 37°C until MEA readings were taken.

We matched the number of neurons plated in each well and between experimental groups. Each plate compared the 3 groups of neurons, prepared, and transfected on the same day, WT and variants, so that they could be analyzed in a head-to-head manner.

MEA plates were read 72 hours after plating in the Axion Biosystems Maestro Multi-Well MEA system (Axion BioSystems, Atlanta, GA). The environment was allowed to equilibrate to 22°C (for consistency with patch-clamp experiments) or 37°C (to determine the mutant effect on neuronal firing at body temperature) and 5% CO_2_ for 5 minutes prior to recording. Spontaneous trigeminal neuron firing activity was recorded for 10 minutes. For experiments with menthol, after measuring spontaneous activity under basal conditions, menthol in culture media was added to a final concentration of 100 µM and additional spontaneous firing activity was recorded for 10 minutes. Only neuron depolarizations (spikes) from active electrodes (defined as >1 spike/minute) were counted. For each well, the number of spikes from active electrodes was counted. This sum was then normalized by the number of active electrodes that generated those spikes. Readings were taken over 10 minutes, so normalized spike counts were divided by the duration of the recording to calculate the firing frequency. The number of active electrodes/neurons from each condition over all biological replicates is reported as n in Results. The mean and standard error of the mean reported for these experiments were calculated from data obtained from all biological replicates; however, each data point on MEA graphs represents individual biological replicates.

### Data Analysis and Statistics

Our data analysis and statistical testing were performed based on the work we have performed and developed in prior studies.^4^ Details: normalization was performed to control the variations in channel expression and inward current amplitude and to be able to fit the recorded data with the Boltzmann function (for voltage-dependence). The Sophion Qube is an automated electrophysiology instrument that performs experimentation and cell selection in a blinded manner. Cell selection is also performed in a randomized manner. Subsequent filtering and analysis of the data are also performed in a nonbiased manner, where automated filters are applied to the entire data set from a given Qube experimental run. The fitting and graphing of the data were performed using Prism 9 software (Graphpad Software Inc., San Diego, CA) (PRISM, RRID:SCR_005375) (GraphPad, RRID:SCR_000306). In our statistical tests, we fit each datum from each cell (e.g., activation) with the relevant equation (e.g., Boltzmann). On completion of the calculations and by using curve fitting, the numbers (e.g., midpoint, V_1/2_) from each of the cells were binned as either WT or variant, as given in reference 4. All necessary measures for sample sizes for each experiment were taken into account. The statistical *p* values report our results obtained from the *t* test: when overall 2 conditions were being compared. A level of significance α = 0.05 was used with *p* values less than 0.05 being considered to be statistically significant. All values are reported as means ± standard error of means (SEM) or errors in fit, when appropriate, for *n* recordings/samples. In our figures, we display SEM because it indicates the range in which the mean would deviate if the experiment were to be completely repeated. Our distributive data are plotted as scatter plots displaying individual data points in relevant figures. The declared group size is the number of independent values (number of biological replicates), and the statistical analysis was performed using these independent values, as given in reference 4.

### Data Availability

All information needed to assess the findings of this study is contained within the article. Additional data can be obtained from the corresponding author on request.

## Results

### Patient Characteristics

**Figure 1:**
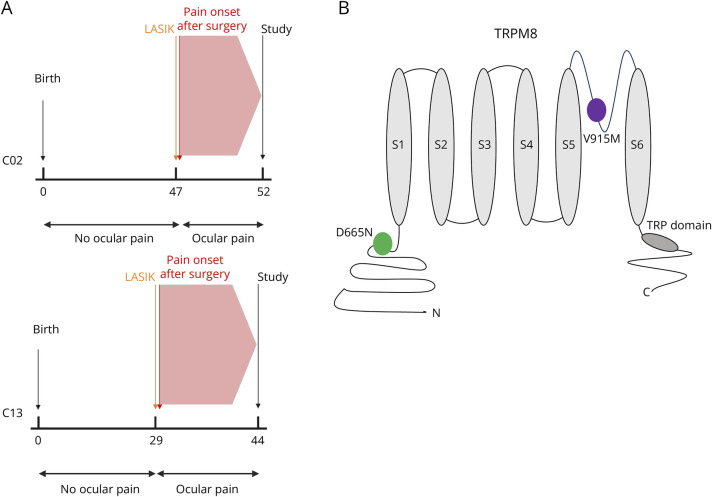
Clinical Course and Locations of the Mutant Variants of TRPM8 (A) Timeline of pain phenotype development for patients. The X-axis indicates time in years, and the Y-axis indicates events. (B) Location of the mutations on a generic TRPM8 schematic. D665N indicated in green, and V915M is indicated in purple.

These mutations were identified in 2 patients who had previously undergone LASIK surgery. The first patient, a male (C02), 52 years old at the time of study, had LASIK performed in both eyes at age 47. Pain and eye dryness developed immediately after surgery, persisted, and was present throughout the day, with only partial relief from multiple medications. His OSDI was 37.5 at the time of study (Figure 1A). The second patient, also a male (C13), 44 years old at the time of study, had LASIK performed in both eyes at age 29. Pain symptoms began a few weeks after surgery. The patient experienced persistent, continuous pain in both eyes, as well as eye dryness; moisture chamber goggles provided only partial relief. His OSDI was 50, when assessed at the age of 44 (Figure 1A). The D665N (C02) mutation (single–amino acid substitution) is located on the N-terminus of the channel, and the V915M (C13) mutation is located on the pore loop (Figure 1B).^25,26^

### Biophysical and Pharmacologic Characterization

**Figure 2:**
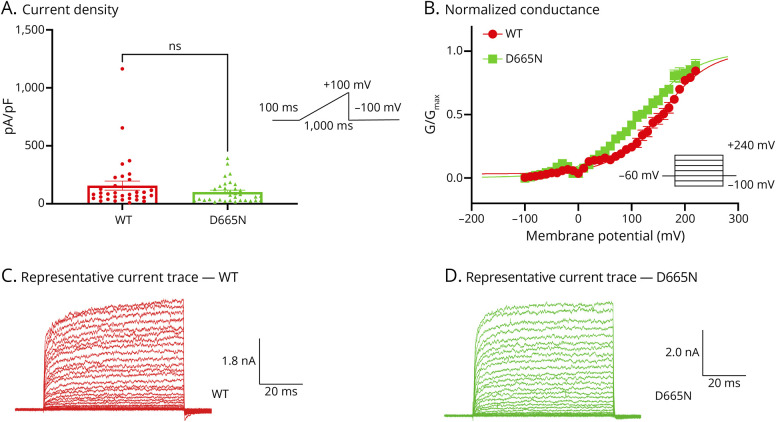
D665N Left-Shifts the Activation Curve (A) Current density of WT vs D665N shown in pA/pF obtained using a ramp protocol. (B) Activating curves displayed as normalized conductance as a function of membrane potential. (C and D) Representative sample macroscopic currents elicited by patch-clamp recordings. The pulse protocols are shown as insets. Data are means ± SE. NS = not significant; WT = wild-type.

**Figure 3:**
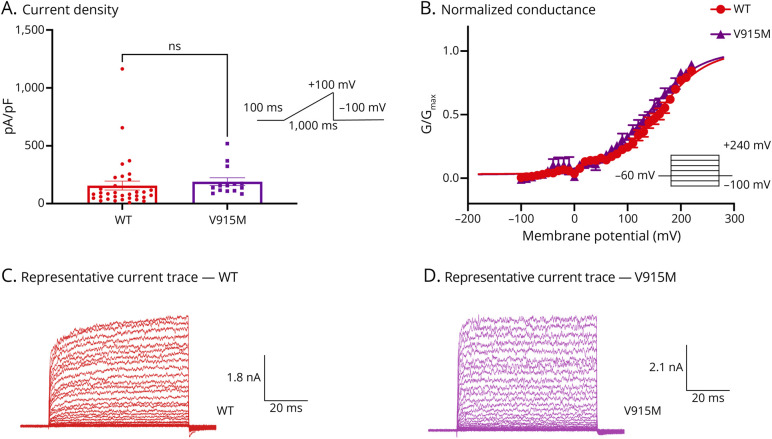
V915M-Tendency Toward Left-Shift in the Activation Curve, Not Statistically Significant (A) Current density of WT vs V915M shown in pA/pF obtained using a ramp protocol. (B) Activating curves displayed as normalized conductance as a function of membrane potential. (C and D) Representative sample macroscopic currents elicited by patch-clamp recordings. The pulse protocols are shown as insets. Data are means ± SE. NS = not significant; WT = wild-type.

To characterize baseline properties of D665N and V915M compared with WT channels, we performed patch-clamp experiments. All patch-clamp experiments were conducted in a head-to-head (simultaneous) manner, using our automated high-throughput patch-clamp instrument. We first used a ramp protocol in which we held cells for 100 ms at −100 mV, followed by a ramp stimulus to +100 mV over the timespan of 1,000 ms, which was followed by a pulse back down to −100 mV. Using this protocol, we measured current amplitude from each variant and divided this number by the cell capacitances providing the maximal current density in cells transfected with WT vs mutant channels (Figures 2 and 3). Our results suggest that there are no significant differences in basal maximal current density between either channel variant vs WT at +100 mV (*p* > 0.05) (Figures 2A and 3A).

Next, we measured steady-state currents during voltage steps from −100 mV to +240 mV from a holding potential of −60 mV.^22^ Normalized conductance was plotted as a function of membrane potential and fitted with a Boltzmann function. Our results indicate that the midpoint (V_1/2_) of the activation curve for D665N was significantly shifted in the leftward direction (*p* = 0.0371) vs WT (WT = 156.1 ± 2.0 mV, D665N = 119 ± 2.4 mV, n = 5–14). There was a leftward shift of the V_1/2_ of activation for the V915M mutant channel (V915M = 138.9 ± 2.7 mV, n = 9); however, this change was not statistically significant (*p* > 0.05) (Figures 2, B–D and 3, B–D).

**Figure 4:**
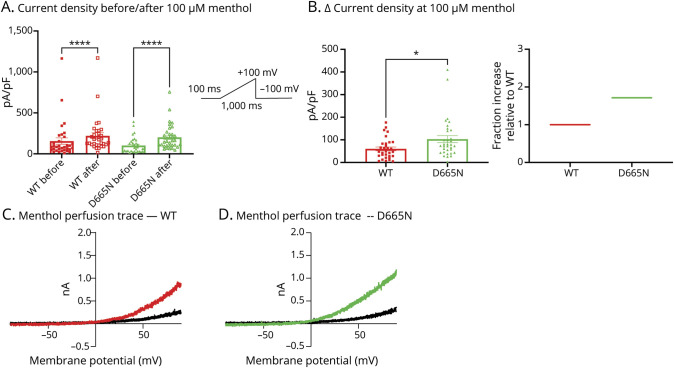
Menthol Sensitivity Is Increased in D665N vs WT Sensitivity of the WT and D665N mutant channels was assessed in a matched-paired manner. (A) Current density as pA/pF before and after menthol perfusion at 100 µM. Pulse protocol is shown as inset. (B) Delta (∆) current density comparison in response to menthol between variants. On the right, fractional increase relative to WT channels. (C and D) Representative current traces shown as current-voltage relationships, where the traces are characterized by reversal potential near 0 mV. Data are means ± SE. * Indicates statistical significance. WT = wild-type.

**Figure 5:**
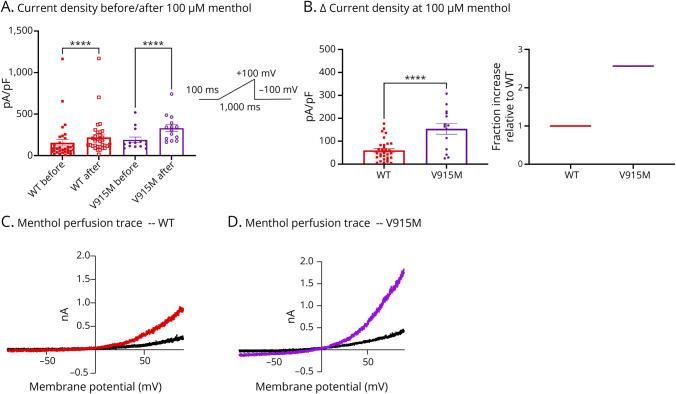
Menthol Sensitivity Is Increased in V915M vs WT Sensitivity of the WT and V915M mutant channels were assessed in a matched-paired manner. (A) Current density as pA/pF before and after menthol perfusion at 100 µM. Pulse protocol is shown as inset. (B) Delta (∆) current density comparison in response to menthol between variants. On the right, fractional increase relative to WT channels. (C and D) Representative current traces shown as current-voltage relationships, where the traces are characterized by reversal potential near 0 mV. Data are means ± SE. * Indicates statistical significance. WT = wild-type.

We also tested the response to menthol, a well-known agonist for TRPM8, at a concentration of 100 µM ^13^ in WT vs mutant variants (Figures 4 and 5). Expectedly, after perfusing menthol onto cells transfected with each of the 3 variants, the current densities significantly increased as measured at +100 mV (*p* < 0.0001) (Figures 4A and 5A). Because an increased sensitivity to menthol is considered a gain-of-function effect in TRPM8,^13^ we subtracted each channel variants' basal current density from their response to menthol (measured as ∆). Our results show that both D665N (*p* = 0.0245) and V915M (*p* = 0.0002) produce significantly larger responses to menthol than WT (Figures 4, B–D and 5, B–D). Collectively, our biophysical and pharmacologic analysis of these channels shows that both mutations impart net gain-of-function attributes on TRPM8 channels.

### D665N and V915M Increase Trigeminal Ganglion Neuron Excitability

**Figure 6:**
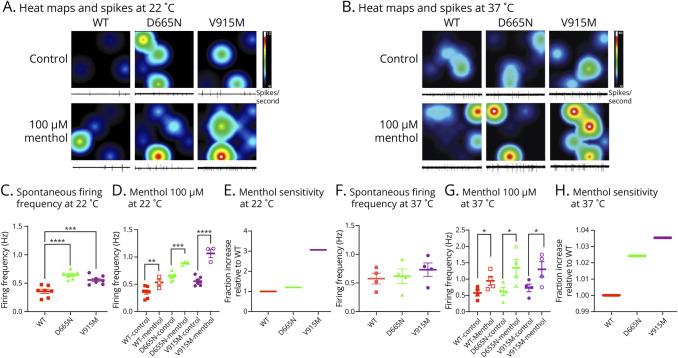
Excitability of Trigeminal Ganglion Neurons Is Increased by Expression of Mutant vs WT at 22 and 37°C MEA was used to assess the firing of trigeminal ganglion neurons expressing WT or mutant channels. (A and B) Representative heat maps and spike traces from MEA recordings of trigeminal ganglion neurons expressing each of the 3 TRPM8 variants. The top row shows spontaneous activity under control conditions for WT, D655N, and V915M, while the bottom row shows spontaneous activity in the presence of 100 µM menthol. Each square is from an individual well of the 24-well plate, with a 4 × 4 array of electrodes at the base of the well. The right panel shows the scale of firing frequency (spikes/second), from 0 (black/blue) to 10 (white). Each circle represents an active electrode within a 4 x 4 electrode array. The interelectrode distance is approximately 350 µm. Under each heatmap is a representative firing trace from a single electrode within the array displayed, at 22°C. (C) Spontaneous firing frequencies between WT and mutant. Each data point is the normalized firing frequency of a single replicate. The mean and SEM are also displayed for each condition, at 22°C. (D) Menthol pharmacology at 100 µM, at 22°C. (E) Fraction of trigeminal ganglion neuronal spontaneous firing in response to menthol vs WT, at 22°C. (F–H) Same as C–E, but at 37°C. Data are means ± SE. * Indicates statistical significance. Cnt = control; WT = wild-type.

To determine whether the mutant channels increase the excitability of trigeminal ganglion neurons, we used MEA recordings. Similar to our patch-clamp experiments, these MEA recordings were also conducted in a head-to-head manner.^4^ Plasmids encoding WT, D665N, and V915M TRPM8 channels were individually electroporated into sister cultures of trigeminal ganglion neurons on the same day. Spontaneous firing rates of the neurons expressing WT, or each of the 2 mutations, were measured over the time course of 10 minutes at the temperatures of 22 and 37°C (Figure 6). The first set of recordings at 22°C (same temperature at which patch-clamp recordings were conducted) were consistent with patch-clamp recordings. We found that both D665N (firing frequency = 0.646 ± 0.026 Hz, n = 183, *p* = 0.0104) and V915M (firing frequency = 0.547 ± 0.035 Hz, n = 208, *p* = 0.0385) significantly increased trigeminal ganglion neuron firing frequency relative to WT (firing frequency = 0.356 ± 0.038 Hz, n = 155) (Figure 6C). Next, to determine the effects of menthol on neuronal firing, we measured spontaneous activity in the presence of 100 µM menthol (similar to patch-clamp experiments). Our results indicated that consistent with the patch-clamp recordings (Figure 6, D and E), neuronal firing was increased in menthol vs control (firing frequencies, WT = 0.533 ± 0.60 Hz, D665N = 0.886 ± 0.0159 Hz, and V915M = 1.06 ± 0.079 Hz). The trends in increased excitability in response to menthol, as determined by a fractional increase in spontaneous firing relative to WT (Figure 6E), were also consistent with ∆ current density (Figures 4B and 5B) (WT: *p* = 0.0079; D665N: *p* < 0.0001; V915M: *p* = 0.0006).

To determine mutant effects on spontaneous firing of trigeminal ganglion neurons at core body temperature, we conducted MEA recordings at 37°C. While there were not statistically significant increases in neuronal excitability in neurons expressing the D655N (firing frequency = 0.617 ± 0.127 Hz, n = 257) and V915M (firing frequency = 0.731 ± 0.117 Hz, n = 241) mutants compared with WT (firing frequency = 0.575 ± 0.093 Hz, n = 244) (Figure 6F) (*p* > 0.05), there were significant increases in excitability in response to menthol (firing frequencies, WT = 0.948 ± 0.138 Hz, D665N = 1.343 ± 0.263 Hz, and V915M = 1.299 ± 0.234 Hz) (WT: *p* = 0.0336; D665N: *p* = 0.0237; V915M: *p* = 0.0363) (Figure 6G); furthermore, the fractional increase in firing in neurons expressing mutant channels was larger relative to WT (Figure 6H). These results indicate that at the core body temperature of 37°C, when TRPM8 channels are expected to be dormant,^22^ the presence of WT vs either of the mutant channels does not alter neuronal firing, and it is only when menthol (a TRPM8 agonist) is added that the spontaneous activity increases (Figure 6). This effect occurs to a larger extent in mutant channels vs WT even at the core body temperature.

## Discussion

The molecular mechanisms underlying pain arising after axonal injury to peripheral neurons remain incompletely understood, and the basis for interindividual differences in pain after axotomy is not understood. In an effort to address this question, we have investigated the molecular genetic substrates for chronic pain, which occurs in only a small subpopulation of individuals after LASIK surgery which injures corneal axons. In our WES study, the cohort of 21 patients with persistent pain after LASIK identified mutations in multiple genes that comprise the electrogenisome in trigeminal ganglion neurons.^5^ In the absence of phenotype-genotype segregation in multigenerational families, functional testing of these variants is necessary to attribute likelihood of pathogenicity.^27^ Our investigations of a missense mutant of Nav1.7 (P610T), discovered in 2 male siblings with persistent ocular pain after corneal refractive surgery, suggested that a small but proexcitatory destabilization of the slow-inactivated states of Nav1.7, when combined with a secondary insult in the form of axonal transection, could culminate in chronic pain.^4^ Given the well-established role of Nav channels, in particular Nav1.7, within the pain pathway and its contribution to human pain disorders,^28,29^ the discovery of gain-of-function mutations in Nav1.7 in this cohort of patients with persistent pain is not entirely surprising.

By contrast, the role of TRPM8 in pain sensation in humans is not as well-established. A genome-wide association study identified a SNP in TRPM8, which is linked to chronic migraine and allodynia in Han Chinese participants.^30^ However, the functional effect of this SNP on function of the channel is not known. Recently, we identified a gain-of-function mutation in TRPM8 (R30Q) in a patient with trigeminal neuralgia and demonstrated that it confers multiple gain-of-function attributes, i.e., enhanced activation, increased basal [Ca^2+^]_i_, and enhanced menthol response, on the channel.^13^ In this study, we assessed D665N and V915M, 2 mutations in the N-terminus and pore loop of TRPM8, respectively, that were found in patients with persistent ocular pain after corneal refractive surgery. Previous investigations have determined that the N- and C-terminal domains are crucial for proper function, localization, and assembly of the TRPM8 channel into tetramers,^31^ and the pore loop is important for the ion permeation pathway. The pore domain in TRPM8 has also been suggested to be a key molecular determinant in the channel temperature response.^32^

We performed patch-clamp investigations of these mutants at 22°C because TRPM8 becomes activated at cooler temperatures, and thus, we argued that any potential biophysical differences between D665N and V915M vs WT would be most apparent at temperatures when these channels are expected to be active. The results of our study show that D665N significantly shifts the activation curve of TRPM8, indicating a gain-of-function. As menthol is a known pharmacologic tool for activating these channels, we measured the sensitivity of these mutants to this agent. Our results show that both mutant variants conduct significantly larger currents than WT channels, which further indicates a gain-of-function. Our singular macroscopic conductance data from patch-clamp recordings were recapitulated in the MEA recordings at the same temperature, which demonstrated increased excitability of trigeminal ganglion neurons carrying the mutant channels. Expectedly, MEA recordings at core body temperature of 37°C did not demonstrate an increase in excitability in neurons carrying the mutant vs WT channels because TRPM8 is typically activated at temperatures below ∼27°C.^22^ Given that TRPM8 is not expected to be active at 37°C,^22^ this finding is not surprising. However, we found that, at 37°C,^33^ spontaneous activity was increased in the presence of menthol (a TRPM8 agonist) and that the fractional increase in menthol sensitivity was larger in mutant vs WT channels. This raises the question of whether there might be a mechanism whereby an endogenous TRPM8 agonist might contribute to the pathogenesis of pain in these patients (see references 34-39). Given that these mutations were identified in patients with persistent ocular pain after LASIK, it will be interesting to determine whether TRPM8 antagonists could be used therapeutically to restore mutant TRPM8 function.^40^

Multiple studies have implicated TRPM8 in the physiology of the eye. This channel has been implicated as a regulator of tear production and participates in maintenance of ocular surface wetness via its activity as a cold thermoreceptor, which contributes to basal tear flow.^14^ Menthol activation of TRPM8 at lower concentrations induces lacrimation without evoking nocifensive behaviors in rodents, whereas higher concentrations of menthol can induce both lacrimation and nocifensive behaviors.^15^ These findings have resulted in TRPM8 being regarded as a therapeutic target for severe dry eye disease.^41^ Given that LASIK is known to trigger abnormalities of lacrimation^42^ along with pain, the mutations that we have profiled may play a role in both physiologic manifestations in our patients.

**Figure 7:**
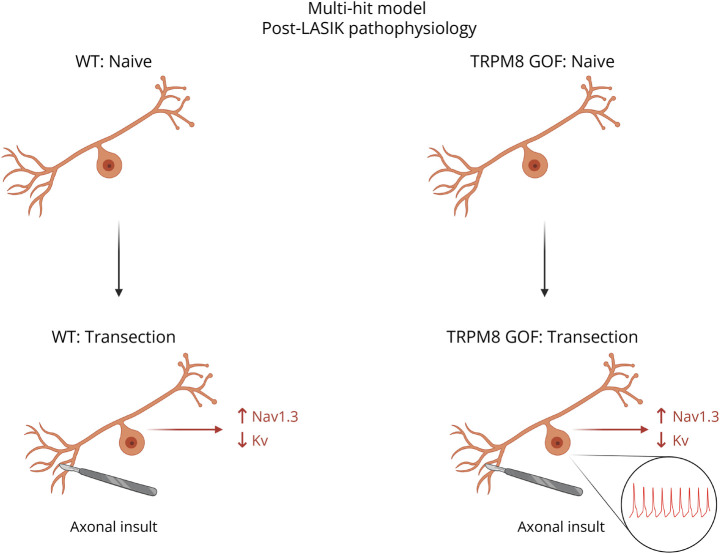
Multihit Model Contributing to Post-LASIK Pathophysiology Axonal transection causes dysregulation in the electrogenisome, such as an upregulation of Nav1.3 and/or downregulation of Kv channels, that produces trigeminal ganglion neuron hyperexcitability in the context of gain-of-function mutations such as D655N and V915M.

In the patients under study, persistent ocular pain occurred following corneal refractive surgery in which distal axons of trigeminal ganglion neurons are transected. In this regard, it is well-established that injury to the peripheral axons of sensory neurons triggers changes in the electrogenisome, such as upregulation of Nav1.3, which contributes to neuronal hyperexcitability.^43-45^ Moreover, peripheral axotomy triggers downregulation of Kv channels that serve to stabilize and prevent firing of these cells, thus adding another proexcitatory change.^45^ Indeed, previous studies have suggested that the balance between Kv1 and TRPM8 channels is critical to the excitability of trigeminal cold-sensing neurons under both injured and uninjured conditions.^46,47^ We speculate that, in the case of patients with WT genes within their electrogenisome, dysregulatory changes such as upregulation of Nav1.3 and/or downregulation of Kv channels are not sufficient to produce pain, while they trigger a degree of hyperexcitability that produces pain in the context of D665N and V915M mutant channels (Figure 7). This idea, of a combinatorial effect of changes in expression of multiple channels, should be investigated in future studies.

Collectively, our findings in this study suggest that proexcitatory mutations of TRPM8, in the context of axonal injury within the cornea, can produce persistent ocular pain. In addition to providing additional evidence for a role of TRPM8 in human pain, our results suggest that inhibitors of this channel merit future study.

## Appendix Authors

**Table:**

Name	Location	Contribution
Mohammad-Reza Ghovanloo, PhD	Department of Neurology, Yale University School of Medicine, New Haven; Center for Neuroscience and Regeneration Research, Yale University; Neuro-Rehabilitation Research Center, Veterans Affairs Connecticut Healthcare System, West Haven, CT	Drafting/revision of the manuscript for content, including medical writing for content; major role in the acquisition of data; study concept or design; analysis or interpretation of data
Philip R. Effraim, MD, PhD	Center for Neuroscience & Regeneration Research, Yale University; Neuro-Rehabilitation Research Center, Veterans Affairs Connecticut Healthcare System, West Haven; Department of Anesthesiology, Yale University School of Medicine, New Haven, CT	Drafting/revision of the manuscript for content, including medical writing for content; major role in the acquisition of data; study concept or design; analysis or interpretation of data
Sidharth Tyagi, MD, PhD	Department of Neurology, Yale University School of Medicine, New Haven; Center for Neuroscience and Regeneration Research, Yale University; Neuro-Rehabilitation Research Center, Veterans Affairs Connecticut Healthcare System, West Haven, CT	Major role in the acquisition of data
Alecia M. Aldrich, BSc	Department of Neurology, Yale University School of Medicine, New Haven; Center for Neuroscience and Regeneration Research, Yale University; Neuro-Rehabilitation Research Center, Veterans Affairs Connecticut Healthcare System, West Haven, CT	Major role in the acquisition of data
Xiaoyang Cheng, PhD	Department of Neurology, Yale University School of Medicine, New Haven; Center for Neuroscience and Regeneration Research, Yale University; Neuro-Rehabilitation Research Center, Veterans Affairs Connecticut Healthcare System, West Haven, CT	Major role in the acquisition of data
Jun-Hui Yuan, MD, PhD	Department of Neurology, Yale University School of Medicine, New Haven; Center for Neuroscience and Regeneration Research, Yale University; Neuro-Rehabilitation Research Center, Veterans Affairs Connecticut Healthcare System, West Haven, CT	Major role in the acquisition of data
Betsy R. Schulman, PhD	Department of Neurology, Yale University School of Medicine, New Haven; Center for Neuroscience and Regeneration Research, Yale University; Neuro-Rehabilitation Research Center, Veterans Affairs Connecticut Healthcare System, West Haven, CT	Major role in the acquisition of data
Deborah S. Jacobs, MD	Department of Ophthalmology, Massachusetts Eye and Ear, Harvard Medical School, Boston	Major role in the acquisition of data; analysis or interpretation of data
Sulayman D. Dib-Hajj, PhD	Department of Neurology, Yale University School of Medicine, New Haven; Center for Neuroscience and Regeneration Research, Yale University; Neuro-Rehabilitation Research Center, Veterans Affairs Connecticut Healthcare System, West Haven, CT	Drafting/revision of the manuscript for content, including medical writing for content; analysis or interpretation of data
Stephen G. Waxman, MD, PhD	Department of Neurology, Yale University School of Medicine, New Haven; Center for Neuroscience and Regeneration Research, Yale University; Neuro-Rehabilitation Research Center, Veterans Affairs Connecticut Healthcare System, West Haven, CT	Drafting/revision of the manuscript for content, including medical writing for content; study concept or design; analysis or interpretation of data

## Glossary

HEK: human embryonic kidney
Kv: voltage-gated potassium channel
LASIK: laser-assisted in situ keratomileusis
MEA: multielectrode array
Nav: voltage-gated sodium channel
TRPM8: transient receptor potential melastatin 8
WES: whole-exome sequencing
WT: wild-type
